# New features in MEK retinopathy

**DOI:** 10.1186/s12886-018-0861-8

**Published:** 2018-09-14

**Authors:** Pallavi Tyagi, Cynthia Santiago

**Affiliations:** Department of Ophthalmology, Aberdeen Royal Infirmary, Foresterhill Road, Aberdeen, AB25 2ZN, UK

**Keywords:** MEK retinopathy, Molecular chemotherapy, MEK inhibitors, BRAF inhibitors, Ocular toxicity

## Abstract

**Background:**

The use of molecularly targeted therapy is becoming widespread in oncology. These agents cause tumour-specific genetic alterations in signal transduction pathways, hence less generalised toxicity. Dabrafenib, a BRAF inhibitor and Trametinib, a MEK inhibitor are two molecularly targeted agents recently approved for treatment of advanced, unresectable melanomas. MEK retinopathy is a recently introduced term describing retinal toxicity secondary to MEK inhibitors.

**Case presentation:**

A 71-year-old man presented with ‘circular, green patches’ in his central vision for 2 weeks. He had multiple relapsed stage IV BRAF gene mutant malignant melanoma. He was on treatment with Dabrafenib *(Tafinlar)* for 7 months and Trametinib *(Mekinist)* for 4 months respectively. The fundus looked normal. The OCT scan showed bilateral symmetrical cystoid macular edema, intraretinal and subretinal fluid, thickening of elliposoid zone and subretinal granular deposits. The symptoms resolved with temporary cessation of chemotherapy but OCT signs persisted.

**Conclusion:**

This case report identifies two new remarkable features of MEK retinopathy as thickening of ellipsoid zone and ‘starry sky’ pattern of distribution of subretinal granular deposits. These changes signify photoreceptors/ RPE toxicity and dysfunction. The subretinal granular deposits showed increased autofluorescence suggested abnormal lipofuscin clearance due to RPE dysfunction. The molecularly targeted therapy has revolutionized the cancer treatment and increased the survival rate. These agents are relatively new and recently approved for clinical use and most of them are associated with ocular toxicities. Awareness of ocular symptoms, side-effect profile of drugs, monitoring regime and liaison between oncologist and eye care professional with ocular imaging is key to early diagnosis and management of ocular adverse events.

## Background

Anticancer therapy as chemotherapeutic agents, hormonal and molecular targeted treatments can produce ocular toxicity. The ocular adverse events (AE) can occur due to effect on cellular proliferation, disruption of ocular immune privilege or direct toxicity to ocular structures. Whereas conventional chemotherapy targets both normal and rapidly dividing cells, newer agents tend to exploit tumour-specific genetic alterations in signal transduction pathways.

Almost 50% of malignant melanomas have somatic mutation of BRAF gene [1]. BRAF inhibitors act on enzyme B-Raf, which plays a role in the regulation of cell growth. Dabrafenib is a BRAF inhibitor that was approved by National Institute of Clinical Excellance (NICE) in United Kingdom (UK) for treating unresectable or metastatic BRAF V600 mutation-positive melanoma in 2014. Another molecular pathway commonly affected in malignant melanomas is mitogen-activated protein kinase/ extracellular regulated kinase (MAPK/ERK) also known as MEK pathway. Trametinib is a MEK inhibitor that inhibits MEK1 and MEK2 genes. Clinical trial data demonstrated that resistance to Dabrafinib and other BRAF inhibitors occurs within 6 to 7 months [2]. This resistance can be overcome by the combination of BRAF inhibitor Dabrafenib with the MEK inhibitor Trametinib [2–4]. Trametinib in combination with Dabrafenib for treating unresectable or metastatic melanoma was approved by NICE in UK in 2016.

Whereas Dabrafenib disturbs ocular immune privilege and is commonly associated with uveitis, Trametinib and other MEK inhibitors usually develop retinal toxicity considered as ‘class effect’ [5, 6]. ‘MEK retinopathy’ is an umbrella term used to describe the dose- and time- dependent retinal side-effects observed with MEK inhibitor therapy [5, 6]. MEK inhibitor clinical trials have reported ocular toxicities in 5–38% of treated patients. [7]. The wide range of incidence may be due to the lack of uniformity in the description, diagnosis and reporting of the same condition, differences in potency of MEK inhibition, schedule of administration and the frequency of ophthalmologic assessment across trials [5].

## Case presentation

**Fig. 1:**
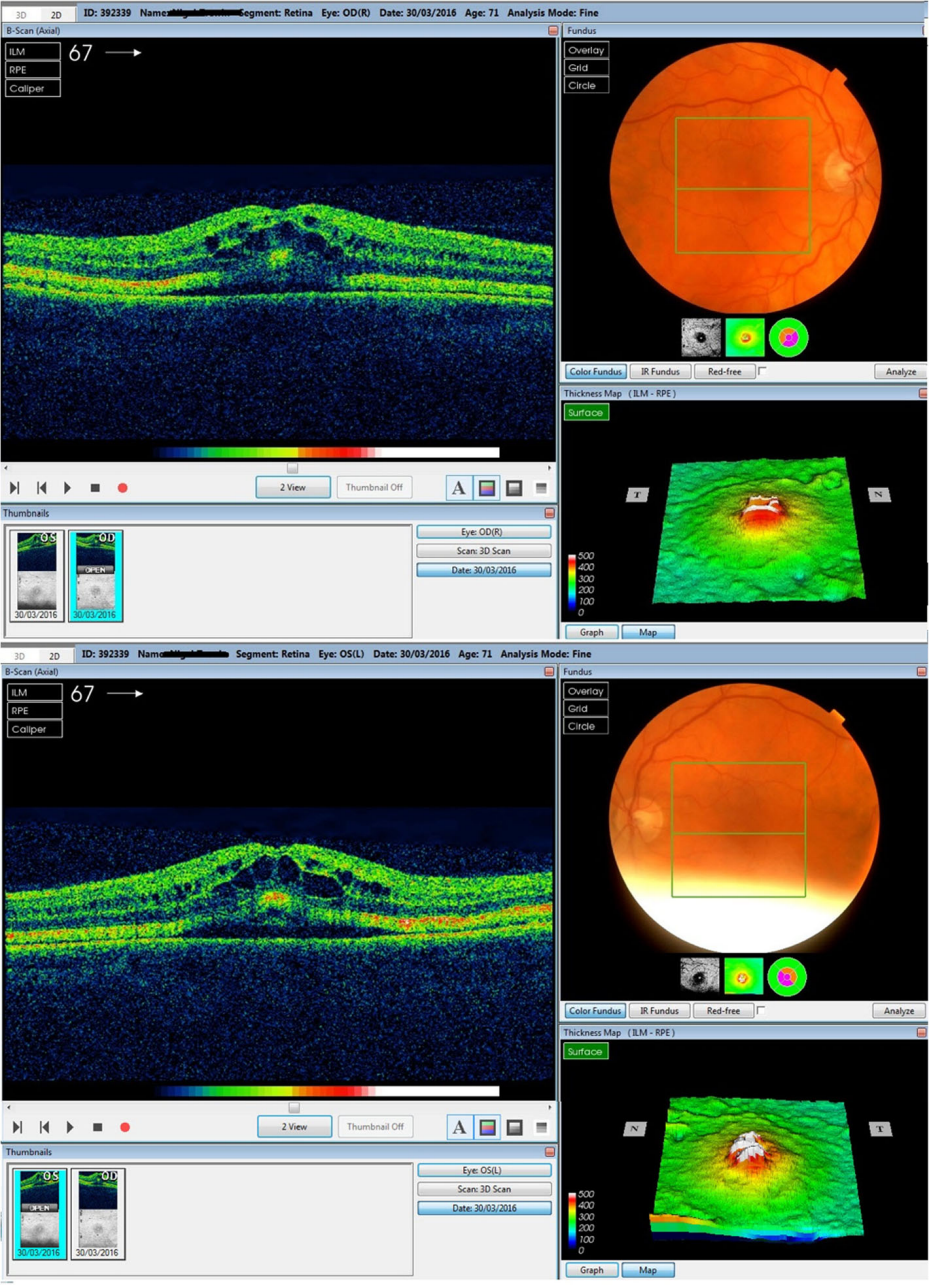
OCT image at presentation: Bilateral cystoid macular edema at presentation

**Fig. 2:**
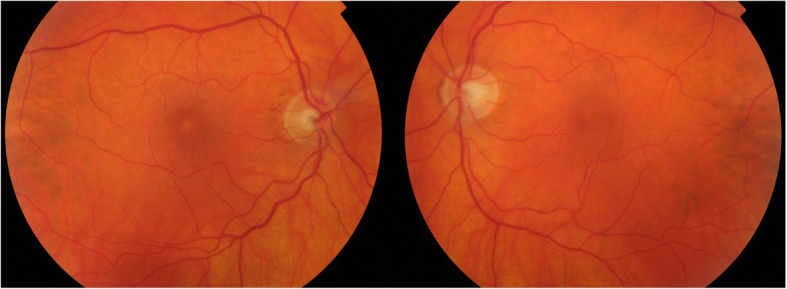
Fundus appearance: Healthy optic disc, dull foveal reflex and normal vasculature

**Fig. 3:**
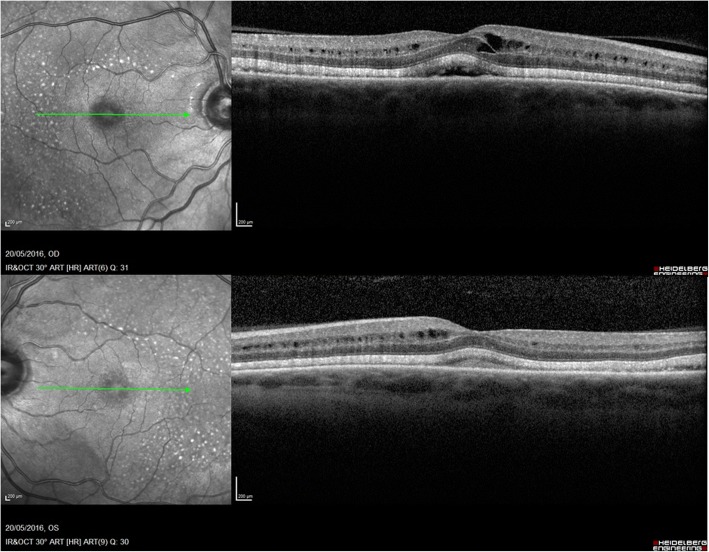
OCT images 5 days after stopping treatment: Intra-retinal and sub-retinal fluid, thickening of ellipsoid zone, sub-retinal deposits and multiple hyper-fluorescent spots on IRR image

**Fig. 4:**
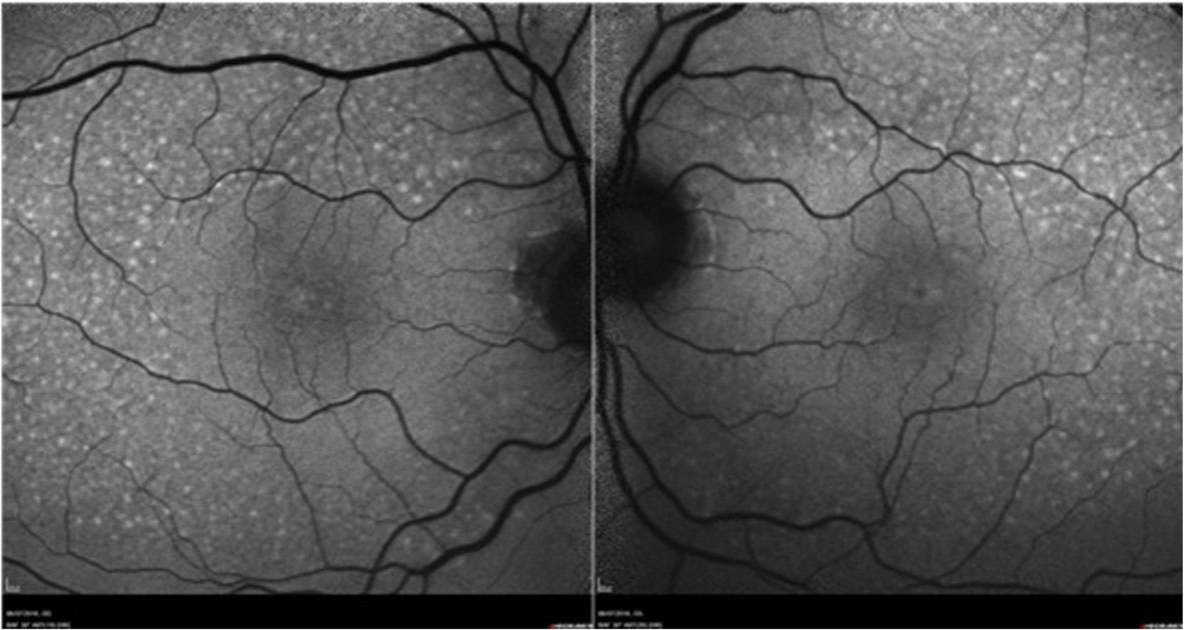
Autofluorescence image: Increased autofluorescence of scattered sub-retinal granular deposits in macular area

A 71-year-old presented to community optometrist with ‘patchy vision’ and ‘green circular patches’ in central vision of both eyes for 2–3 weeks. He was noted to have bilateral cystoid macular edema on optical coherence tomography (OCT) scan and was referred to hospital eye services (Fig. 1). The patient had previously received annual ophthalmic assessments and retinal imaging by optometrist which were unremarkable. He had been seen in eye clinic with left sided herpes zoster ophthalmicus disciform keratitis and uveitis 7 months previously with no retinal involvement. His medical history included hypertension and multiple relapsed stage IV BRAF gene mutant malignant melanoma involving skin, liver and spleen for 11 years. The melanoma nodules were present on buttock, back and periumblical region. He had been treated with surgical excision of nodules, inguinal nodal and femoral vein resection and radiotherapy with consequent lymphoedema of lower limb over years. His most recent melanoma relapse was 15 months previously and was treated with 3 cycles of Ipilimumab which were later discontinued due to uncontrolled diarrhoea. Subsequently, he was found to have enlargement of periumbilical and liver melanoma nodules with appearance of a new nodule in the lung. At this point he was commenced on molecularly targeted oral chemotherapeutic agents, Dabrafenib *(Tafinlar)* 150 mg twice a day (BD) initially followed by Trametinib *(Mekinist)* 2 mg once a day (OD) for 7 months and 4 months respectively. Since the molecularly targeted therapy is known to have ocular side-effects the patient was given comprehensive side-effect profile information at the initiation of treatment and was advised to seek immediate ophthalmic assessment if any visual disturbance. This prompted him to see his optometrist at the onset of symptoms. In view of the ocular symptoms, the oncology team advised him to stop his chemotherapeutic drugs, Dabrafenib and Trametinib and requested specialist ophthalmology review. He was seen in eye clinic 5 days after stopping treatment. His symptoms were marginally better. The visual acuity was 6/6 in both eyes on Snellen’s chart. The anterior segments were normal. Fundus examination showed healthy optic discs, macula showed dull foveal reflex with normal periphery and retinal vasculature (Fig. 2). The OCT scan showed intra-retinal and sub-retinal fluid on macula with cystoid changes in the peri-foveal area. More strikingly, there was significant thickening of the ellipsoid zone and sub-retinal granular deposits overlying an intact looking retinal pigment epithelium (RPE). Infrared reflectance image (IRR) showed multiple hyper-reflective lesions that corresponded to the sub-retinal granular deposits on OCT imaging. These deposits were distributed in bilateral symmetrical ‘starry sky’ appearance on the macula (Fig. 3). The choroid appeared unaffected with choroidal thickness of 180 μm in right eye and 225 μm in left eye. On autofluorescence (AF) imaging, the macular sub-retinal granular lesions showed increased autofluorescence (Fig. 4). The macular edema looked improved compared to scans sent by community optometrist.

**Fig. 5:**
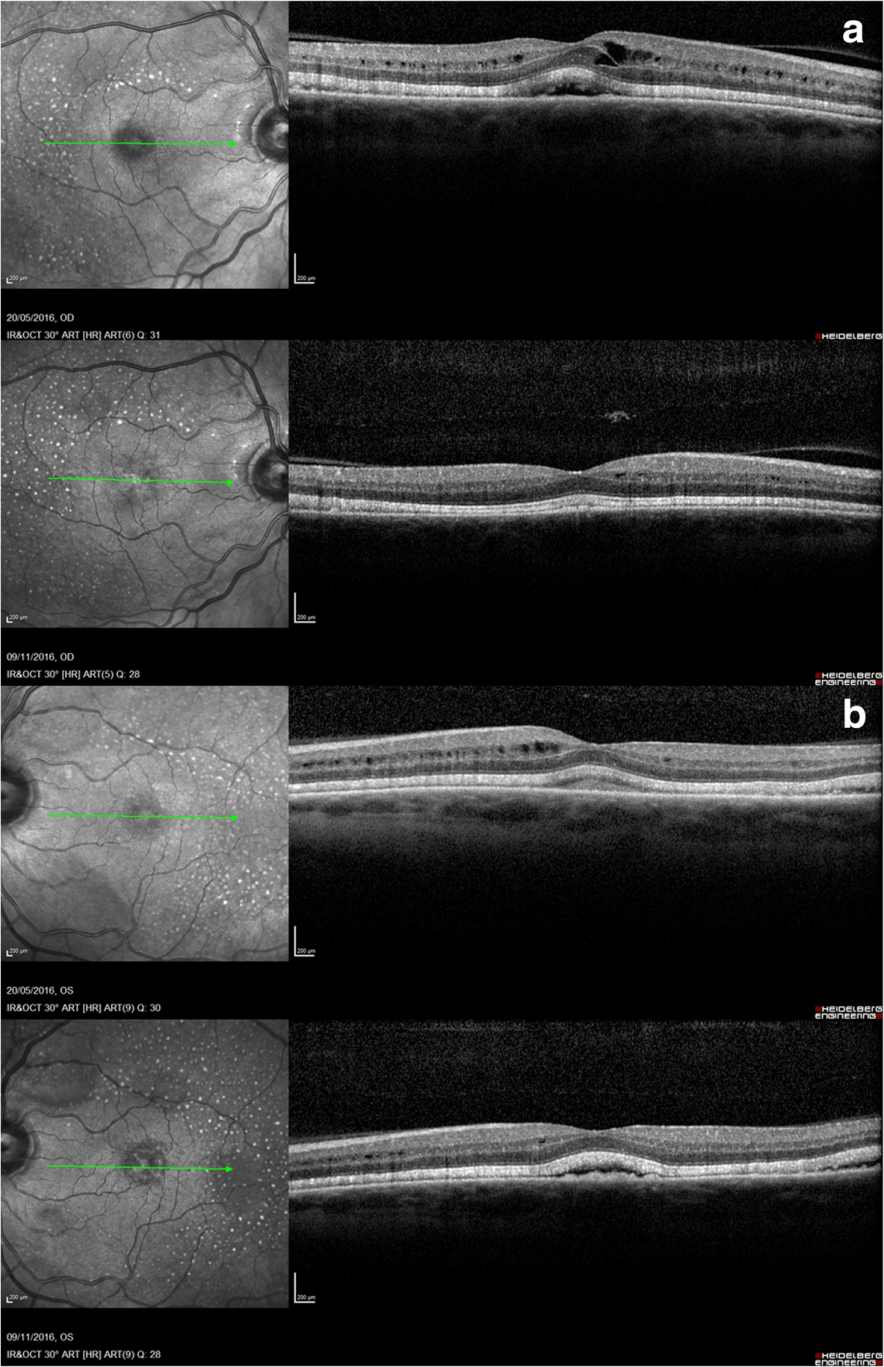
Change in OCT appearance in both eyes over 6 months: Resolution of cystoid macular edema in perifoveal region, reduction in the intra-retinal and sub-retinal fluid. The ellipsoid zone thickening and subretinal granular deposits remained unchanged in right (**a**) and left (**b**) eye

His chemotherapeutic drugs, Dabrafenib and Trametinib were withheld for 3 weeks. He noticed subjective improvement in symptoms with clearing of dark patches in his vision within 10 days. His vision, fundus and OCT appearances were unchanged. He was recommenced on reduced dose of Dabrafinib (100 mg) BD and Trametinib (1 mg) OD after 3 weeks to prevent melanoma relapse. He remained visually asymptomatic and clinically unchanged on this dose for 6 months. Over time, the cystoid macular edema resolved in the perifoveal region with reduction in the intra-retinal and sub-retinal fluid. The ellipsoid zone thickening and subretinal deposits remained unchanged (Fig. 5). Unfortunately, the patient’s melanoma progressed and he was awaiting further chemotherapy with Pembroluzimab.

## Discussion and conclusion

MEK retinopathy usually presents acutely within the first week of the first dose. The retinal features described in MEK retinopathy include central serous retinopathy, serous retinal detachment, cystoid macular edema, intra-retinal fluid and cysts and thin choroid. Most of these features are identified on optical coherence tomography scans [6–10]. The retinopathy is typically bilateral and symmetrical [5–9]. In cases where only one eye is affected, other diagnoses should be considered [5]. Symptoms of MEK retinopathy can vary from being asymptomatic to blurred vision, altered color perception, shadows, light sensitivity, metamorphopsia and glare. Cases are often mild, short-lived, self-limiting, and do not interfere with activities of daily living [8, 11–13]. Central retinal thickness and volume showed dose-dependent increases after the start of treatment, followed by a marked decrease despite continued treatment [8]. The retinopathy partially recovers, but can still be detected many months later [12]. Retinal thinning and retinal atrophy have been observed after long-term treatment [14] Cessation of life-extending treatment with MEK inhibitors is not indicated when SRF is present [12].

MEK pathway and its activation by the fibroblast growth factor receptor (FGFR) plays prominent role in the maintenance, survival and repair of RPE. Inhibition of this pathway leads to degeneration of RPE cells. The pathophysiology of MEK retinopathy is due to acute RPE toxicity which results in RPE hyperpermeability and breakdown of the retinal–blood barrier [15–17].

The treatment of MEK retinopathy is based on Common Terminology Criteria for Adverse Events (CTCAE) criteria, widely used for AE reporting in oncology studies, include a 4-category grading scheme for retinopathy according to symptom severity [18]. Asymptomatic patients and mild retinopathy with vision better than 6/12 do not require interruption of dosing as mild symptoms and OCT abnormalities frequently resolve within days after continued dosing. This suggests that many patients with MEK retinopathy develop tachyphylaxis to continued MEK inhibitor therapy. For significant visual symptoms or vision below 6/12, patients should be instructed to interrupt dosing with MEK inhibitor therapy. When symptoms resolve, patients may be rechallenged at the same dose of MEK inhibitor therapy with close monitoring following re-initiation of treatment. For toxicities with severe visual impairment or interruption of daily activities, MEK inhibitor treatment should be discontinued and when symptoms and OCT findings resolve, patients may be rechallenged at a lower dose [5, 18].

Our case showed bilateral, symmetrical changes within the retina in the form of cystoid edema, intra-retinal fluid and sub-retinal fluid signifying abnormal RPE permeability. Two new features were identified as thickening of ellipsoid zone and characteristically distributed subretinal granular deposits. The thickening of the ellipsoid zone could be due to swelling of the photoreceptors secondary to RPE toxicity and dysfunction affecting the photoreceptor nutrition. The subretinal granular deposits overlying a normal appearing RPE showed increased autofluorescence suggested abnormal lipofuscin clearance due to RPE dysfunction. Although the intra-retinal and sub-retinal fluid reduced, the ellipsoid layer changes and the granular changes persisted signifying ongoing RPE dysfunction despite reduced dosage even though the patient became asymptomatic. We were unable to perform fundus fluorescein angiography and electrodiagnostic tests which could have potentially shed more light on the structural and functional changes in retina.

In this case report we identified two new features of MEK retinopathy not previously described in literature. Introduction of molecularly targeted therapy have revolutionized the cancer treatment and increased the survival rate. Most of these agents are associated with ocular toxicities. These agents are relatively new and recently approved by NICE for clinical use in cancer treatment. Awareness of ocular symptoms, side-effect profile of drugs, monitoring regime and liaison between oncologist and eye care professional with ocular imaging is key to early diagnosis and management of ocular adverse events.

## Abbreviations

AE: adverse event
MAPK/ERK, MEK: National Institute of Clinical Excellance: NICE mitogen-activated protein kinase/ extracellular regulated kinase
BD: twice a day
OD: once a day
OCT: optical coherence tomography
RPE: retinal pigment epithelium
AF: autofluorescence
FGFR: fibroblast growth factor receptor
CTCAE: Common Terminology Criteria for Adverse Events
